# Ultrasound detection of prostatic calculi as a parameter to predict the appearance of hematospermia after a prostate biopsy

**DOI:** 10.1590/S1677-5538.IBJU.2016.0005

**Published:** 2017

**Authors:** Lucio Dell'Atti

**Affiliations:** 1 Department of Urology, University Hospital “St. Anna”, Ferrara, Italy

**Keywords:** Prostate, Ultrasound, High-Intensity Focused, Transrectal, Biopsy, Hemospermia

## Abstract

**Purpose:**

We evaluated the correlation between prostate calculi and hematospermia in patients undergoing prostate biopsy, and its impact on sexual activity of patients.

**Materials and Methods:**

A single-center prospective randomized study of 212 patients referred for transrectal ultrasound-guided prostate biopsy (TRUSBx) was performed. All patients were divided into two groups: Group A (GA), 106 patients with moderate/marked presence of prostatic calculi visualized by TRUS; Group B (GB), 106 patients with absence/scarce of prostatic calcifications. Patients were handed questionnaires to obtain a validated data on the duration and impact of hematospermia on sexual activity. The anxiety scores were recorded using a visual analogue scale.

**Results:**

No significant difference was noted between the two groups when comparing age, preoperative PSA level, prostate volume, and biopsy number, except for digital rectal examination (DRE) findings. Post-biopsy results of patients included in GA revealed that the complication of hematospermia was present in 65.1%, while in GB was present in 39.7% (p<0.001).

On multivariate analysis for identifying significant preoperative predictors of hematospermia, which included variables of age, PSA, prostate volume, and prostate cancer were not shown to be significant predictors of hematospermia, except DRE and prostate calculi (p<0.001).

The mean anxiety score was 3.7±2.8 in GA and 2.3±1.9 in GB, respectively (p<0.001).

**Conclusions:**

Prostatic calculi are an independent predictive factor of severe hematospermia after TRUSBx on the basis of multivariate analysis, but don't affect the positive rate of prostate cancer. Patients should be adequately counselled before TRUSBx to avoid undue anxiety and alterations in sexual activity.

## INTRODUCTION

Prostatic calculi are presumed to form by the precipitation of prostatic secretions and calcification of the corpora amylacea under inflammatory conditions (1, 2). Prostatic calculi are common in men who are evaluated for benign prostatic hyperplasia or prostate cancer (3) and are discovered incidentally, usually by means of ultrasound investigation for other medical conditions. The study of prostate with transrectal ultrasound (TRUS) provides both axial and sagittal images and thus improves the evaluation of the number, location, and dimension of the prostatic calculi. Transrectal ultrasound-guided prostate biopsy (TRUSBx) is one of the most common urological procedures, approximately 1 million prostate biopsies are performed each year in Europe (~650.000) and United States (~350.000) as diagnostic investigation of patients with clinical suspicion of prostate cancer (4). TRUSBx is generally a safe procedure with minimal haemorrhagic complications. However, hematospermia is a well-recognized complication of TRUSBx (18-31%) (5). Although it is classified under minor complication, its persistence causes distress to the patient and the partner in sexual activity (6). In this study we prospectively evaluated patients undergoing prostate biopsy for suspicion of prostate cancer, what effect prostatic calculi will have on appearance of hematospermia after prostate biopsy, and its impact on sexual activity of patients.

## MATERIALS AND METHODS

A single-center prospective randomized study of 212 consecutive patients referred for TRUSBx to our Department was performed between May 2010 to November 2015.

All patients underwent an initial TRUSBx for abnormal digital rectal examination (DRE), high prostate-specific antigen (PSA) levels (≥4ng/mL), or both. Patients with a history of biopsy, on anticoagulation/antiplatelet therapy, surgical treatment of prostatic disease, neoadjuvant therapy, or no sexually active men were excluded from our study. No patient had any history of bleeding disorders. None had any history of hematospermia within 2 years before. Patients were instructed to take antibiotics, usually levofloxacin 500mg orally, for 5 days starting the evening before the procedure and a small evacuative enema starting two hours before the procedure. All patients enrolled in the study signed a consent form for the procedure. TRUSBx was performed with the patient in the left lateral decubitus using a General Electric Logiq 7 machine (GE Healthcare, Milwaukee, WI, USA) equipped with a 5-9MHz multi-frequency convex probe “end-fire”. Each TRUS performed included an assessment of the prostatic diameter, the volume of the whole prostate, the transition zone, capsular, seminal vesicle characteristics, presence/absence of prostatic calcification, and a morphological description of potential pathological features. The prostate volume was invariably calculated using prostate ellipse formula (0.52 x length x width x height).

All patients were divided into two groups, as it follows: Group A (GA) included 106 patients with moderate/marked presence of prostatic calculi visualized by TRUS; Group B (GB) included 106 patients with absence/scarce of prostatic calcifications.

**Figure 1:**
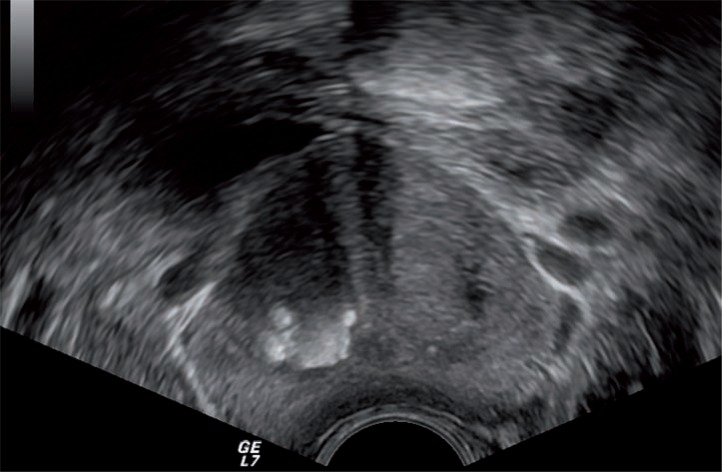
Transrectal ultrasound in axial section of prostate shows marked calcifications with multiple hyperechoic foci and with significant area (≥3mm) with coarse shadow.

**Figure 2:**
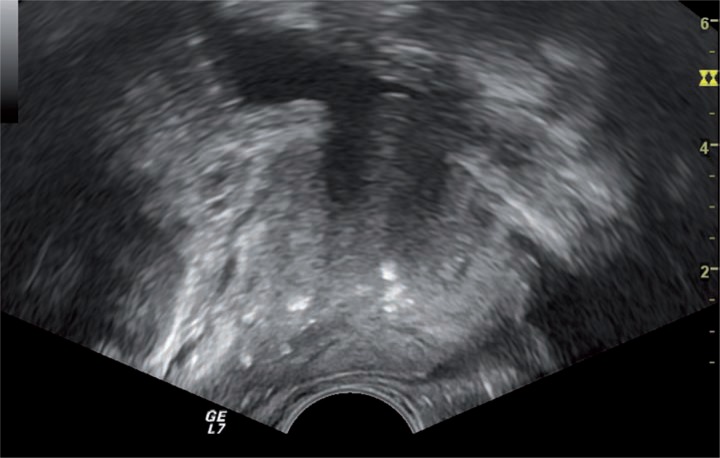
Transrectal ultrasound in longitudinal section of prostate shows mild calcifications with 1 or multiple small foci without coarse shadow.

We defined moderate/marked presence of prostatic calculi as multiple (≥3 in number) hyperechoic foci, with significant area (≥3mm in the largest diameter) with coarse shadow detected in both dimensions (Figure-1). Mild calcifications were defined as 1 or multiple small foci without coarse shadow (Figure-2). All measurements were made by an experienced urologist. After having images of the prostate, sampling was carried out with a 18-Gauge Tru-Cut (Bard Biopsy Systems, Tempe, AZ, USA) needle powered by an automatic spring-loaded biopsy disposable gun. A 14-core biopsy scheme was performed in each patient, as first intention, including 2 basal samples (lateral and medial), 2 parasagittal samples (lateral and medial), 2 apical samples (lateral and medial), and 1 transitional zone sample on each side.

The patients were treated under local anaesthesia with Lidocaine Spray 10gr/100mL (ECO-CAIN^®^, Molteni Dental, FI, Italy) applied two minutes before the procedure (6). All patients received a detailed information guide before the procedure and a questionnaire where information of the participant's age, PSA, prostate volume (PV), DRE findings, use of anticoagulants/antiplatelet, and the number of cores biopsy taken were recorded. The patients were called about 20 days after the procedure to deliver histopathological examination. In this time, we gave to the patients a questionnaire to obtain data on post-biopsy experience. Patients were asked questions about complications occurred (yes/no), how many times (hours/days), severity of hematospermia on a scale 0-4, which was designed with 0 representing absence of bleeding and 4 severe bleeding (5). The presence or absence of each bleeding for its duration and severity was reported but amount was not quantified. Values of 1-2 were classified as low severity and values of 3-4 were classified as high severity. Patients were handed questionnaires to obtain a validated data on the duration and impact of hematospermia on emotions and sexual activity. The anxiety scores were recorded using: 0-no anxiety, 10-extreme anxiety.

### Statistical analysis

Comparisons between the two groups were performed using the Mann-Whitney U test for continuous variables and the chi-square test or Fisher's exact test for categorical variables. Univariate logistic regression analysis was used to identify the individual clinical factors predictive of appearance of hematospermia. A p<0.05 was considered to indicate statistical significance.

## RESULTS

The mean±standard deviation age of enrolled patients was 62.4±6.8 years, with a prostate PV of 47.5±19.9mL, initial PSA of 7.2±5.8ng/mL. The number of biopsy cores was 10.7±4.5. No significant difference was noted between the two groups when comparing age, preoperative PSA level, prostate volume, and biopsy number, except for DRE findings. In Group A more frequent abnormal DRE findings were observed (Table-1). In GA, 3 patients (2.9%) received 5/6 core biopsies, 8 patients (7.5%) received 7/8 core biopsies, 11 patients (10.4%) received 9/10 core biopsies, 16 patients (15.1%) received 11/12 core biopsies, 43 patients (40.5%) received 13/14 core biopsies, and 44 patients (23.6%) received 15/16 core biopsies. In Group B (GB), 4 patients (3.8%) received 5/6 core biopsies, 9 patients (8.5%) received 7/8 core biopsies, 8 patients (7.5%) received 9/10 core biopsies, 13 patients (12.3%) received 11/12 core biopsies, 49 patients (46.2%) received 13/14 core biopsies, and 23 patients (21.7%) received 15/16 core biopsies. Prostate cancer was detected in 74 patients (34.9%) and their Gleason score were ≤6 (42 patients, 56.7%), 7 (23 patients, 31.1%), and ≥8 (9 patients, 12.2%). Post-biopsy results of the 212 patients included in GA revealed that the complication of hematospermia was present in 65.1% (69/106) of patients, while in GB was present in 39.7% (42/106) of patients (p<0.001).

**Table 1:** Demographic and clinicopathologic features of patients undergoing transrectal prostate biopsy.

Characteristics		Group A (n:106)	Group B (n:106)	p value
Age (years), mean ± SD		63.2±6.2	62.7±6.9	NS
PSA (ng/mL), mean ± SD		7.1±5.9	7.8±5.2	NS
PV (mL), mean ± SD		46.8±19.5	47.4±19.3	NS
Abnormal DRE, n (%)		43 (40.5)	28 (26.4)	<0.001
N° biopsy cores, mean ± SD		10.6±4.9	10.9±4.3	NS
Prostate cancers, n (%)		38(35.8)	36(33.9)	NS
**Gleason score, n (%)**				NS
	≤6	23(60.5)	19(52.7)	
	7	10(26.3)	13(36.2)	
	≥8	5(13.2)	4(11.1)	
Hematospermia, n (%)		69(65.1)	42(39.7)	<0.001

**SD =** standard deviation; **PSA =** prostate-specific antigen; **DRE =** digital rectal examination; **PV =** prostate volume; **NS =** not significant

In Table-2 shows the incidence and severity of hematospermia between the two groups. Grade 1, 2, 3 and 4 complications occurred in 27 (24.3%), 33 (29.7%), 34 (30.6%), and 17 (15.3%) patients, respectively. Two patients in GA were admitted to hospital having developed urinary retention. The study revealed significant difference in the incidence in terms of hematospermia (p<0.002) and its severity (p<0.001) between both groups. Moreover, a statistically significant difference was found in the duration of hematospermia between two groups. Forty-two patients ejaculated in the first week. The duration was longer in GA (16.9 days) than in GB (11.3 days; p<0.002). The number of patients still reporting hematospermia at 4 weeks after TRUSBx was 12 (17.4%) in GA, and 4 (9.5%) in GB (p<0.001). On multivariate analysis for identifying significant preoperative predictors of hematospermia, which included variables of age, PSA, PV, and prostate cancer were not shown to be a significant predictor of hematospermia, except DRE and prostate calculi (p<0.001) (Table-3).

**Table 2:** Incidence of complications among the two groups.

	Group A (n:106)				Group B (n: 106)				Total
Complication grade	G1	G2	G3	G4	G1	G2	G3	G4	
Hematospermia	9	18	27	15	18	15	7	2	111

**Table 3:** Results of multiple logistic regression analysis examining the correlation between clinicopathological variables and hematospermia risk after prostate biopsy.

Characteristics	Adjusted Odds ratio	95% CI	p value
Age	0.703	(0.251-1.843)	NS
PSA	1.314	(0.614-3.132)	NS
Prostate Volume	1.22	(0.542-3.102)	NS
Abnormal DRE	2.128	(0.703-3.691)	<0.001
Prostate calculi	3.461	(2.308-5.624)	<0.001
Prostate cancer	2.015	(0.601-3.142)	NS

**PSA =** prostate-specific antigen; **DRE =** digital rectal examination; C**I =** confidence interval; **NS =** not significant.

The mean anxiety score was 3.7±2.8 in GA and 2.3±1.9 in GB, respectively (p<0.001). Forty-nine patients in both groups reported less sexual activity due to hematospermia, its duration and recurrence.

## DISCUSSION

One of the most frequent and embarrassing complications of TRUSBx is hematospermia. Hematospermia is the presence of blood in seminal fluid (4). It is almost always caused by nonspecific inflammation of the prostate and seminal vesicles. In patients without coagulopathy, the incidence of this complication varies with patient's factors as prostate size, anticoagulant medications, and procedural factors such as the number of biopsy cores taken (7). Hematospermia can cause extreme concern and anxiety in the patients and their sexual partners (8).

Prostatic calculi are commonly diagnosed by TRUS and their incidence is believed to begin after puberty and increase with age (9). TRUS is safe, cost-effective, radiation free and an excellent imaging modality for the prostate and seminal vesicles. Several articles reported on the findings of TRUS in patients with prostatic calcifications (10–12). At TRUS, prostatic calculi appear as well circumscribed focal foci of increased echogenicity with or without posterior acoustic shadowing, situated in the prostate gland or seminal tract (12). The mechanism of prostatic calculi formation is unknown. Proposed contributing factors include infections, urinary retention or reflux into the prostate, penetration of spermatozoa into prostatic glands, and desquamation of prostatic epithelium (13). Prostatic calculi have also been showed in association with various systemic diseases, prostatic diseases, and treatment modalities. Examples include hyperparathyroidism, hypercalciuria, prostatic hypertrophy and carcinoma, status after radiotherapy, and status after adenomectomy or transurethral prostate resection (14, 15). To our knowledge this study is the first to evaluate the effect of prostatic calculi on the outcome of TRUSBx in terms of appearance of hematospermia and its impact on emotions and sexual activity in patients. Gu M et al. (16) reported that patients with prostatic calculi, after the prostate biopsy, experienced more uncomfortable feelings and enjoyed higher urinary retention probability. Most studies have been showed that the degree of urinary retention may be relative to the presence of large prostatic calculi (2, 3, 17). Ludwing et al. (18) concluded that prostatic calculi are typical signs of inflammation. Their study showed a significant difference in the duration of symptoms of chronic prostatitis between prostatic calculi and non-calculi groups, but did not show a significant difference in the white blood cells count in the prostatic secretions. Abdelkhalek et al. (19) referred that after biopsy, the bacteria in calcifications may be disseminated by biopsy needle, then produced local inflammation, further quickly leading to more severe edema. The local inflammation and more severe edema can cause hematospermia. However, unlike the previous studies, in our analysis we have attributed homogeneity in the method of enrollment patients. We prospectively evaluated and enrolled a cohort of patients using an uniform protocol. For one thing, there were no significant differences in the clinical variables among the patients in the two groups, except for DRE findings. In Group A more frequent abnormal DRE findings were observed (p<0.001), probably also due to an increase in the thickness or abnormal shape of glandular prostatic tissue associated with the greatest number of prostatic calcifications. There was not a lack of measuring the sizes and numbers or locations of prostatic calculi when TRUS was performed because the criteria for classifying calcifications have been well established. Moreover, the rate of hematospermia has been calculated from the number of sexually active patients who pledged in sexual activity after TRUSBx and not from the overall number of patients included in the study. Nevertheless, this study had some limitations. The patients were recruited from a single center, the majority of the participants are white, therefore results might not be generalizable to other races. There may be different incidences of prostatic calculi with different distribution in diverse populations. In one autopsy study, the incidence was 70.1% and 29.1% in black men from USA and Nigeria, respectively. They suggest that dietary pattern was an important factor for determining the incidence of prostatic calcifications (20). In addition, although the prostatic secretions cultures were more likely to be positive in patients with prostatic calculi in the present study, the number of those men was unknown, and hence we did not separately assess if positive prostatic secretion culture had an impact on their hematospermia. In fact, Zhao et al. (21) reported in a study of 358 patients that prostatic calcifications are significantly associated with the presence of positive prostatic secretions cultures and erectile dysfunction in chronic prostatitis syndrome males. We also suggested that prostatic calcifications are common and not associated with any significant pathology as prostate cancer (p=0.326). Although some publications as Suh et al. (22) and Smolski et al. (23) showed that interface calcification is common and not associated with any particular pathology, peripheral zone calcification appears to be strongly associated with prostate cancer. However, further studies are required to assess if peripheral prostatic calculi are directly associated with an increased incidence of prostate cancer.

## CONCLUSIONS

Hemospermia is a well-recognized complication of TRUSBx and is mostly self-limited. Although it is classified under minor complications, its persistence causes immense distress to the patient and the sexual partner. In this study we showed that prostatic calculi are an independent predictive factor of severe hematospermia after TRUSBx on the basis of multivariate analysis, but don't affect the positive rate of prostate cancer. This study result would be useful for predicting the uncomfortable feeling before prostate biopsy. Patients should be adequately counselled before prostate biopsy to avoid undue anxiety and alterations in sexual activity.

## Abbreviations

TRUS: = Transrectal ultrasound
TRUSBx: = Transrectal ultrasound prostate biopsy
DRE: = Digital rectal examination
PSA: = Prostate-specific antigen
GA: = Group A
GB: = Group B
VSA: = Visual analogue scale
